# Exploring Gluten Assessment in Marketed Products through a Sandwich ELISA Methodology Based on Novel Recombinant Antibodies

**DOI:** 10.3390/foods13091341

**Published:** 2024-04-26

**Authors:** Eduardo Garcia-Calvo, Aina García-García, Santiago Rodríguez, Rosario Martín, Teresa García

**Affiliations:** Department of Nutrition and Food Sciences, School of Veterinary Sciences, Universidad Complutense de Madrid, 28040 Madrid, Spain; edugar01@ucm.es (E.G.-C.); santro03@ucm.es (S.R.); rmartins@ucm.es (R.M.); tgarcia@ucm.es (T.G.)

**Keywords:** food control, gluten, recombinant Fab, celiac disease, sandwich ELISA

## Abstract

This study presents the development of a sandwich ELISA method for gluten detection in foods, using recombinant Fab antibody fragments against gliadin. The Fabs were chemically biotinylated and immobilized on streptavidin-coated plates as capture antibodies, while alkaline phosphatase-conjugated Fabs were used as detection antibodies. Four different gliadin-binding Fabs were tested and the Fab pair Fab8E-4 and Fab-C showed the best compatibility. An indirect sandwich immunoassay, using unmodified Fab8E-4 for capture and Fab-C as the detection antibody, achieved a detection limit of 26 ng/mL of gliadin, corresponding to 10 mg/kg of gluten in foods. No cross-reactivity was observed against 60 gluten-free species commonly used in the food industry. Analysis of 50 commercial products demonstrated consistent results compared to the standard method for gluten detection. The complete lack of cross-reactivity of the developed immunoassay with oat products potentially provides an advantage over other gluten detection systems.

## 1. Introduction

Gluten, an ethanol-soluble protein fraction found in grains, constitutes a substantial portion, often up to 90%, of wheat proteins [1]. Specific components of gluten, present in various grain species such as wheat, rye, and barley, can trigger adverse effects when consumed by individuals with particular sensitivities. Gluten consumption has been associated with several diseases, classified based on their etiopathology [2]. The most extensively studied gluten-related disorder is celiac disease, an autoimmune process with a prevalence of 1% in Western countries [3].

A gluten-free diet is the most efficient and economically viable treatment for gluten-related disorders. It not only arrests disease progression but also fosters tissue healing [4]. This situation has prompted the establishment of stringent labeling regulations aimed at ensuring the provision of gluten-free diets for individuals allergic or intolerant. Within the European Union, any food product containing gluten must bear clear labeling on its packaging. Such labeling should indicate the presence of gluten in the product, and it must include the precise terms “contains gluten” or “contains cereals containing gluten” within the ingredients list or in a dedicated allergen statement. Furthermore, to qualify for the designation of “gluten-free,” a food product must not exceed 20 parts per million (ppm) of gluten content, while “very low gluten” labeling is permissible when the gluten content remains below 100 ppm [5].

Immunoassays offer unparalleled relevance in the context of gluten detection in food products when compared to alternative analytical methodologies. Their sensitivity, specificity, and efficiency in detecting gluten, even at very low concentrations, underscore their importance. Immunoassays, including enzyme-linked immunosorbent assays (ELISAs) and lateral flow devices, employ specialized antibodies designed to target specifically gluten proteins. Furthermore, immunoassays yield rapid results and are often more cost-effective than alternative methods like polymerase chain reaction (PCR) or mass spectrometry, justifying their widespread adoption across the food industry [6].

Sandwich ELISA offers several distinct advantages in the realm of immunological and biochemical research. One key advantage is its exceptional sensitivity and specificity. By utilizing two antibodies that bind to different epitopes on the target molecule, sandwich ELISA minimizes the potential for false-positive or false-negative results, making it highly reliable for detecting and quantifying specific proteins or antigens [7]. Additionally, this assay format allows for the measurement of proteins in complex mixtures, without interference from other molecules. It provides quantitative data, enabling the precise concentration measurements, robustness, reproducibility, and adaptability that have made it an indispensable tool in various fields, including gluten detection in food, where the most-used methodology is a sandwich ELISA based on a classical monoclonal antibody named R5 [8]. Although classical monoclonal antibodies meant a leap forward in detection of allergens and related molecules, several problems have arisen, mainly derived from the genomic instability of the hybridomas from which the antibodies are derived. Recombinant antibodies have become the perfect alternative to surpass these problems [9]. This work aims to demonstrate the potential of the development and use of recombinant antibodies for gluten detection in foods, a paradigm shift in the field of food science, by presenting the use of two completely novel recombinant antibodies derived direct or indirectly from the celiac immune response, as affinity probes in a sandwich ELISA format [10].

In earlier studies, novel approaches were introduced to develop recombinant antibody fragments, Fabs (fragment antigen binding), for gluten detection in foodstuffs. These approaches involved the construction of two distinct antibody libraries. The first library was a comprehensive immune library derived from lymphocytic RNA [11], while the second library was a hybrid of semi-synthetic heavy chain [12], based on a pre-existing dAb template (an antibody fragment consisting of the variable region of the heavy chain), and immune light chains obtained from the aforementioned immune library. Several Fabs were successfully isolated against gluten through phage display technology and were subsequently produced as recombinant proteins. This process resulted in the obtention of four high-affinity Fabs (Fab-C, Fab-H, and Fab-E from the first library, and Fab8E-4 from the second one), which were characterized for their suitability as probes in ELISA assays, and one of them was tested for its ability to detect gluten in marketed products [13]. The aim of this work was the development of a sandwich ELISA methodology based on those recombinant Fabs, to be used as an alternative method for gluten detection in commercial food products.

## 2. Materials and Methods

### 2.1. Recombinant Fabs Used as Sandwich ELISA Probes

The Fabs (Fab-C, Fab-E, Fab-H, and Fab8E-4) were selected by phage display, expressed in the supernatant of *E. coli* K12 RV308, and purified by IMAC (Immobilized Metal Affinity Chromatography) as previously described [13].

### 2.2. Compatibility Study between the Selected Fabs Using a Direct Sandwich ELISA

The compatibility between the Fabs in a sandwich disposition was checked in a direct sandwich ELISA, by using in vitro biotynilated recombinant Fabs, immobilized in streptavidin plates, as capture antibodies and alkaline phosphatase-conjugated Fabs as detection antibodies.

The biotynilation process started with each Fab dissolved in PBS, which was changed to carbonate/bicarbonate buffer pH 8.3 using an Amicon^®^ ultra-15 centrifugal device (30 kDa MWCO, Merck©, Darmstadt, Germany, ref #UFC903024). The chemical biotinylation was carried out using the EZ-link^®^ kit (Thermo©, Waltham, MA, USA, ref #21363), by adding 37 µg of reactant per Fab milligram and incubating the reaction for 30 min at room temperature. The excess of free biotin was washed away using an Econopac 10 DG column (Biorad©, Hercules, CA, USA, ref #7322010). The eluted fractions containing protein were identified by their absorbance at 280 nm. The biotinylated Fabs were immobilized to streptavidin-coated plates (Thermo©, ref #15122) to be used as capture antibodies.

One milligram of each biotinylated Fab was conjugated with streptavidin–alkaline phosphatase following the manufacturer’s specifications (Merck^®^, ref #S2890) to be used as the detection antibody.

Once the capture antibody was immobilized in the plate, it was blocked with PBS-BSA 3%. The antigens were added, incubated, and washed away. The detection antibody (alkaline phosphatase-conjugated Fab) was then added (0.5 µg/well in the blocking solution), and incubated for 1 h at room temperature. Unbound conjugated Fabs were washed away and, the reveling solution (2 mg/mL 4-nitrophenyl phosphate disodium salt hexahydrate (pNPP) (Sigma©, Sant Louis, MO, USA, ref #N4645) dissolved in diethanolamine-MgCl_2_ buffer (Reagena©, Toivala, Finland, ref #170057)) was added and measured in a spectrophotometer at 405 nm after incubating in darkness for 20 min.

### 2.3. Indirect Sandwich ELISA Protocol

The capture antibodies were affixed directly onto immunosorbent plates (0.5 µg/well of recombinant Fab in PBS) and left to incubate at 37 °C for 1 h. Following this, the coating solution was discarded, and 200 μL of blocking solution (3% BSA in PBS) was applied to each well for 1 h at room temperature. After 10 thorough washes with PBS, the gliadin-PWG or gluten proteins extracted from the samples (following the protocol described in prior research [13]) were diluted in PBS, and 100 µL of antigen dilution were dispensed per well. The plate was then subjected to another 1 h incubation period at 37 ºC. Subsequent to an additional round of washing (10 times with PBS), 0.5 μg of unmodified detection Fab per well, diluted in 100 μL of blocking solution, was added, and incubated for 1 h at room temperature with agitation. Following 15 washes with PBS, the secondary antibody (rabbit anti-human H + L HRP conjugated, Abcam©, Cambridge, UK ref #6759), diluted in 100 μL of blocking solution, was added and incubated for 1 h at room temperature with agitation. After a final washing step (10 times with PBS), 100 μL of Tetramethylbenzidine (TMB) (Sigma©, ref #T0440) was added. The reaction was terminated after 20 min with 50 μL of a diluted sulfuric acid solution, and the signal was recorded at 450 nm.

### 2.4. Assay Validation and Analysis of Commercial Products

The assays used for validating and characterizing the key analytical features of the proposed methodology were essentially as previously described [13]. The limits of detection (LOD) and quantification (LOQ) were calculated by performing a dose-response analysis against a reference material, the gliadin-PWG (prolamin working group) [14]. The goodness of fit was tested using the reduced chi-square parameter. To test specificity for gluten, 60 heterologous species that did not contain gluten and that can be used for the fabrication of gluten-free products were tested to ensure there was no cross-reactivity (Table 1).

A recovery analysis was conducted using gluten-free certified rice flour spiked with solid gliadin-PWG, equivalent to theoretical levels of 20 and 100 parts per million (ppm) of gluten. Additionally, a control was prepared by spiking a rice flour ethanol extract with the same quantities of ethanol-dissolved gliadin-PWG (0.05 µg/mL and 0.25 µg/mL).

Once the analytical features were characterized, 50 commercial food products were analyzed to demonstrate the functionality of the assay in its intended actual application.

The products were sourced from Spanish retailers and categorized based on the information provided on their labels. This comprehensive sampling approach encompassed all possible scenarios, ranging from non-gluten certified products to naturally gluten-containing items such as bakery goods, and products with precautionary labeling, ensuring a thorough analysis of the results.

## 3. Results and Discussion

### 3.1. Selection of Capture and Detection Recombinant Antibodies

The compatibility of different Fab pairs was evaluated using 10 µg/mL of gliadin-PWG as antigen for all combinations. Four gliadin-binding recombinant Fabs (Fab8E-4, Fab-E, Fab-H and Fab-C) obtained in a previous work [13], were evaluated as candidates for the development of a sandwich ELISA for the detection of gluten. To assess compatibility between the four Fabs used to detect gliadin, a checkerboard test was performed, using all the biotinylated Fabs as capture antibodies, and all the alkaline-phosphatase conjugated Fabs as detection antibodies (Table 2).

The combination of biotinylated Fab8E-4 as a capture antibody and Fab-C conjugated with alkaline phosphatase as the detection antibody produced the best results. The same Fab could not be used as the capture and the detection antibody because the absorbance values were lower than expected for the saturating concentration of antigen added. This could be explained due to the competition for the same epitopes in the gliadin molecule when using the same antibodies for capture and detection.

In contrast, the R5 monoclonal antibody is used both as the capture and detection antibody in the sandwich ELISA assay used as reference for gluten detection. That may be due to its ability to recognize repetitive motifs dispersed across gluten proteins, predominantly within the more conserved N-terminal segments of gliadins [15].

Computational models [11,12] indicated that Fab-C and Fab8E-4 exhibit prominent interactions with the gliadin C-terminal regions, albeit in distinct positions (a less conserved region characterized by a reduced occurrence of repetitive motifs). This may explain why pairs of identical Fabs compete for the same epitopes, while combinations of dissimilar Fabs synergistically enhance detection efficiency.

The in vitro biotinylation procedure enabled the conjugation of biotin molecules with up to all available free amino groups. This led to significant alterations in the conformation of the Fabs, resulting in a reduced affinity towards the target [16]. While the direct ELISA protocol adequately assessed Fab compatibility, the biotinylation process introduced a discernible decline in the detection signal. Consequently, the ultimate assay configuration adopted was an indirect sandwich ELISA, employing the Fab pair that exhibited superior detection performance.

### 3.2. Indirect Sandwich ELISA Development and Validation for Gluten Detection

In order to ascertain the selectivity of the sandwich method, an indirect sandwich ELISA methodology was set up and tested with a panel of 60 plant and animal species (Table 1) to evaluate its potential cross-reactivity with matrices devoid of gluten. The outcomes unequivocally highlighted that the sandwich ELISA displayed an absence of cross-reactivity with these ingredients, highlighting the specificity in detecting gluten-like proteins. These results strongly endorse the method as a valuable and precise instrument for gluten immunodetection in food products, as it clearly distinguishes between samples containing gluten and those lacking these proteins.

**Figure 1:**
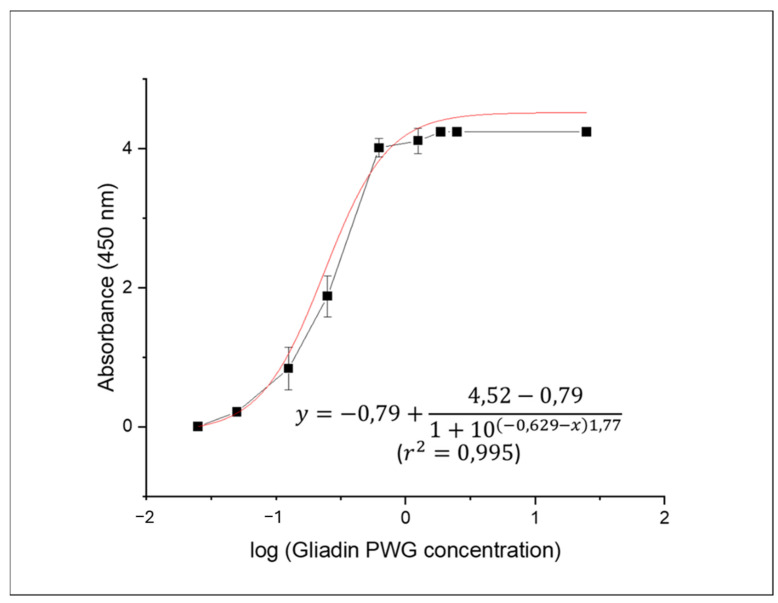
Dose response curve obtained from the analysis of the gliadin-PWG standard (0.025–25 µg/mL) by sandwich ELISA using Fab-C as detection antibody and Fab8E-4 as capture antibody. This model (red line) was used to estimate the gluten content of the tested commercial food products. An HRP-conjugated anti-human Fab was used as the secondary antibody. Mean values of three independent determinations (black squares) and standard deviation of each data set are shown. The reduced chi-square obtained from Origin 8.0 software was χ^2^ = 1.254.

The sensitivity of the indirect sandwich ELISA was assessed through a dose-response model of increasing concentrations of gliadin-PWG, ranging from 0.025 to 25 µg/mL (Figure 1). The limit of detection (LOD) was determined to be 0.026 µg/mL through interpolation, using three times the standard deviation of ten blank samples. This corresponds to a gluten concentration of 10.4 mg/kg in the samples, factoring in a dilution factor of 5, an extraction factor of 40 (0.25 g of sample extracted with 10 mL of buffer), and the conversion factor from gliadin to gluten (two).

Additionally, the limit of quantification (LOQ) was determined by interpolating ten times the standard deviation of the blank samples, resulting in a value of 0.044 µg/mL of gliadin-PWG, which is equivalent to 17.4 mg/kg of gluten. These findings confirm the method’s compliance with the current regulatory limits. Notably, the developed method effectively distinguishes gluten-free products (with less than 20 mg/kg of gluten) from those containing gluten. It is important to note that the sensitivity of this assay is slightly lower than that of the R5 sandwich ELISA, possibly due to variations in assay types and the antibodies used. Unlike the R5 method, which involves two molecular interactions with the antigen, the newly developed indirect sandwich ELISA relies on a single molecular interaction per antigen molecule, facilitated by the Fab’s composition of only one paratope (two in total). This difference allows for a more streamlined approach, with only one binding event per Fab [17]. The results obtained thus demonstrate that the single-paratope molecules (Fab) exhibit a high affinity for the target antigen.

A gluten recovery analysis using the indirect sandwich ELISA was conducted by testing gluten-like proteins (prolamins) extracted from gluten-free certified rice flour spiked with solid gliadin-PWG, equivalent to concentrations of 20 and 100 mg/kg of gluten. The actual gluten content of these mixtures was concurrently assessed using the R5 sandwich ELISA. The results obtained yielded recovery percentages of 104.14 ± 2.2% for the 20 mg/kg mixture and 95.44 ± 6.36% for the 100 mg/kg mixture. To establish a control baseline, a rice flour extract spiked with equivalent quantities of dissolved gliadin-PWG (0.05 μg/mL, corresponding to 20 mg/kg in food samples, and 0.25 μg/mL, equivalent to 100 mg/kg) was prepared. The results for these control samples demonstrated recovery percentages of 100.08 ± 3.67% for the 0.05 μg/mL solution and 95.44 ± 6.36% for the 0.25 μg/mL solution. Accordingly, all the results demonstrated an excellent recovery range close to 100%, affirming the accuracy and reliability of the method [18].

### 3.3. Detection of Gluten in Commercial Samples

The developed methodology was applied for the analysis of gluten-like proteins extracted from a wide variety of commercial food products, encompassing categories such as cereals, dairy, meat, and beverages, spanning both processed and raw items. The collected samples were categorized based on their labeling into four distinct groups: (A) products explicitly indicating the presence of gluten (9 samples); (B) products bearing precautionary labeling stating “may contain gluten” (6 samples); (C) products lacking any declaration of gluten presence or specific gluten warnings (21 samples); and (D) products featuring “gluten-free” labeling and/or certification (14 samples).

The gluten concentration was determined by averaging the absorbance readings from three measurements of each sample and interpolating them on a dose-response curve model. Gluten parts per million (ppm) were calculated considering the previously described dilution process, and the results were categorized as either positive or negative samples in accordance with current European Legislation.

To ensure the precision and reliability of the proposed test, these food samples underwent parallel analysis using the gold standard technique for gluten detection in food, a direct sandwich ELISA with the monoclonal antibody R5. The summarized results are presented in Table 3. Notably, most of the analyzed samples yielded congruent outcomes with both methods, exhibiting a strong alignment with their respective labeling claims. This alignment underscores compliance with the guidelines stipulated by European legislation for classifying food products in terms of gluten content.

The results of 47 out of the 50 products subjected to analysis using the indirect sandwich ELISA were consistent with those obtained from the validation technique, the R5-ELISA. Notably, three products yielded positive outcomes when assessed using the R5 reference assay but registered as negative when evaluated with the proposed methodology. A noteworthy observation was that these particular samples, which deviated from the results of the R5 standard test, contained oats as their primary ingredient. It is postulated that this outcome can be attributed to the lack of cross-reactivity of the recombinant Fabs with oats, potentially explaining the discordance in these instances.

### 3.4. Comparison with Other Immunoassays for Gluten Detection

A direct comparison between the previously developed indirect ELISA, which relies solely on Fab-C, and the newly introduced indirect sandwich ELISA featuring the Fab-C and Fab8E-4 pair, is of significant relevance. On one hand, the indirect approach offers a speed advantage, as it involves one step fewer than the sandwich method. Conversely, the sandwich methodology has demonstrated greater sensitivity, proving a slightly lower limit of detection, as well as a reduced limit of quantification (17 ppm for the sandwich method as opposed to 20 ppm for the indirect method). This can be explained by considering the number of molecular interactions. While both formats allow for a minimum of a single interaction per antigen molecule (avidity), the sandwich format exhibits stronger paratopic affinity due to the provision of two paratope–epitope interactions, in contrast to the singular paratope in the indirect format. A similar occurrence takes place in the sandwich R5 method, but because R5 is a complete antibody molecule, a double interaction per antigen occurs, involving up to four paratopes within the two participating antibodies.

Considering the characteristics of the antigen–antibody interaction, it can be observed that the proposed methodology can achieve adequate detection limits, sufficient for distinguishing commercial products in compliance with European legislation. However, the detection capacity is slightly lower compared to the classical monoclonal antibodies R5 and G12. Nonetheless, these detection levels are obtained using recombinant Fabs, which involve only half the paratopes of a sandwich ELISA, which uses a pair of whole antibodies, indicating a potentially high affinity of the individual paratopes within the developed molecules.

In terms of accuracy, the recovery analysis unveiled notable enhancements with the sandwich format, where the retrieved gluten quantities consistently fell within a narrow range of 95–105% compared to the theoretical concentration. In contrast, the indirect ELISA exhibited a broader variability, with recovery results ranging from 76% to 92%. In addition, this preliminary results showed an incremented accuracy of the proposed Fab-based systems when compared with the R5 sandwich ELISA [8].

Another noticeable feature in both the indirect and sandwich ELISA tests developed is the complete absence of cross-reactivity with oats.

Some authors have found that specific varieties of oats can exhibit cross-reactivity when tested with other detection antibodies such as R5, revealing gluten levels of up to 100 mg/kg in certain oat cultivars [19]. The R5 monoclonal antibody’s cross-reaction can be attributed to the substantial similarity between certain peptides found in avenins (which are gluten-like proteins in oats) and gluten peptides [20]. The absence of this unintentional detection in oat-derived products labeled as gluten-free may indicate the effectiveness of Fab-C and Fab8E-4, making it a valuable tool for evaluating gluten content in such products, especially those containing oat varieties that trigger cross-reactivity with other antibodies available on the market. This characteristic offers a valuable tool in the context of the food industry, to ensure that certified gluten-free oats that are processed in facilities that handle gluten-containing grains are safe for most people with celiac disease, providing a powerful tool to assess cross-contamination in the cereal industry.

The proposed methodology has demonstrated a consistent ability to distinguish gluten-free products among the samples examined.

Identifying gluten in food products remains a significant hurdle in the field of food science. This challenge arises from the fact that gluten is characterized based on its chemical properties, such as solubility in ethanol. Unlike other food allergens, which are typically represented by one or just a few proteins, gluten encompasses a wide variety of proteins. These proteins share certain common characteristics but can vary significantly in their amino acid sequences [21]. This circumstance renders the quest for a universal gluten detection method exceptionally challenging. Numerous comparative investigations have revealed significant disparities among the commercially available kits in the market when it comes to quantifying gluten [22]. These discrepancies can be attributed to variations in the gluten epitopes to which the antibodies bind. For instance, the R5 antibody predominantly identifies epitopes that are more prevalent in rye, barley, or triticale compared to wheat, while the G12 monoclonal antibody demonstrates superior detection capabilities for wheat epitopes [15]. In addition, it has been demonstrated that the quantification of gluten depends on the species and cultivars analyzed, showing significant differences when analyzing the same wheat cultivar with ELISA methods based on polyclonal and monoclonal antibodies such as R5 and G12 [23].

## 4. Conclusions

The use of recombinant antibodies for gluten detection systems provides a more uniform source for standardized affinity probes compared to polyclonal and hybridoma-secreted monoclonal antibodies. Importantly, these advantages are obtained without the necessity of animal involvement. Sandwich ELISA techniques have enhanced the sensitivity and accuracy of single-probe indirect ELISA approaches. Among the four recombinant Fabs exhibiting selective reactivity to gluten, Fab-C and Fab8E-4 have proven not only to be compatible but also to show superior detection capabilities when employed in the direct sandwich ELISA method. Utilizing the Fab-C and Fab8E-4 pair in an indirect sandwich ELISA enables the detection of gliadin concentrations, reaching levels as low as 26 ng/mL, equivalent to 10 mg/kg in food samples. Importantly, this method exhibits no cross-reactivity with any of the analyzed non-gluten-containing species. Furthermore, the developed ELISA test complies with legislative requirements for identifying gluten-free products and offers an advantageous feature not present in some commercial tests, as it does not exhibit cross-reactivity with oat proteins. The novel sandwich ELISA using the recombinant Fab-C and Fab8E-4 is a sensitive, accurate, specific methodology for the detection of gluten in foods, with enhanced proprieties compared to other systems already in the market and could constitute an alternative methodology for assuring food safety to gluten-sensitive patients.

The continuation of this work involves testing the capabilities of analyzing a broader range of samples in interlaboratory trials to ensure method reproducibility. The studied Fabs may find applications as probes in various analytical methodologies, including biosensors. Furthermore, antibody engineering processes could be explored to enhance sensitivity, potentially reformatting the Fabs into whole antibodies (biparatopic and bispecific).

## Figures and Tables

**Table 1:** List of heterologous species (common name and scientific name) tested to assure the absence of cross-reactivity.

Adzuki bean (*Vigna angularis)*	Okra (*Abelmoschus esculentus*)
Almond (*Prunus dulcis*)	Olive (*Olea europaea*)
Arugula (*Eruca vesicaria* subsp. *sativa*)	Onion (*Allium cepa*)
Atlantic salmon (*Salmo salar*)	Papaya (*Carica papaya*)
Avocado (*Persea americana*)	Passion fruit (*Passiflora edulis*)
Beetroot (*Beta vulgaris*)	Pea (*Pisum sativum*)
Blackberry (*Rubus fruticosus*)	Peanut (*Arachis hypogaea*)
Button mushroom (*Agaricus bisporus*)	Pepperomia (*Peperomia pellucida*)
Cabbage (*Brassica oleracea*)	Persimmon (*Diospyros kaki*)
Cantaloupe (*Cucumis melo* var. *cantalupensis*)	Pigeon pea (*Cajanus cajan*)
Cape gooseberry (*Physalis peruviana*)	Plum (*Prunus domestica*)
Cashew (*Anacardium occidentale*)	Quince (*Cydonia oblonga*)
Cassava (*Manihot esculenta*)	Raspberry (*Rubus idaeus*)
Castor bean (*Ricinus communis*)	Red banana (*Musa acuminata*)
Cherry tomato (*S. lycopersicum cerasiforme*)	Rice (*Oryza sativa*)
Chickpea (*Cicer arietinum*)	Runner bean (*Phaseolus coccineus*)
Chili pepper (*Capsicum annuum*)	Scotch bonnet pepper (*Capsicum chinense*)
Chinese cabbage (*Brassica rapa* subsp. *pekinensis*)	Sesame (*Sesamum indicum*)
Common bean (*Phaseolus vulgaris*)	Soybean (*Glycine max*)
Cucumber (*Cucumis sativus*)	Spinach (*Spinacia oleracea*)
European strawberry (*Fragaria vesca)*	Strawberry (*Fragaria x ananassa*)
Ginger (*Zingiber officinale*)	Strawberry tree (*Arbutus unedo*)
Grape (*Vitis vinifera*)	Sunflower *(Helianthus annuus)*
Leek (*Allium ampeloprasum*)	Sweet almond (*Prunus dulcis var. dulcis*)
Lemon (*Citrus limon*)	Tabasco pepper (*Capsicum frutescens*)
Lentil *(Lens culinaris)*	Teff *(Eragrostis tef)*
Mandarin orange (*Citrus reticulata*)	Tomato (*Solanum lycopersicum*)
Mango (*Mangifera indica*)	Vanilla (*Vanilla planifolia*)
Mung bean (*Vigna radiata*)	Walnut (*Juglans regia*)
Myrtle (*Myrtus communis*)	Watermelon (*Citrullus lanatus*)

**Table 2:** Checkerboard compatibility test between pairs of recombinant Fabs in a direct sandwich ELISA. The values represent average absorbance data (405 nm) in three independent assays.

		Detection Fab			
		Fab8E-4	Fab-E	Fab-H	Fab-C
Capture Fab	Fab8E-4	1.254	0.612	0.412	4.741
	Fab-E	0.841	0.523	0.341	0.745
	Fab-H	0.647	0.389	0.415	0.714
	Fab-C	2.751	0.748	0.477	1.784

**Table 3:** Results obtained for the detection of gluten in commercial food products using the developed indirect sandwich ELISA and the sandwich monoclonal R5 antibody for result confirmation.

Products	Number of Items	Sandwich Fab ELISA	R5
(A) Items labeled as gluten containing (9)			
Noodles	2	+(2)	+(2)
Rice products	3	+(3)	+(3)
Oat products	1	+(1)	+(1)
Cereals	2	+(2)	+(2)
Milk-derived products	1	+(1)	+(1)
(B) Items labeled with “may contain” warning (6)			
Cereal	4	+(3)/−(1)	+(3)/−(1)
Flakes	2	−(2)	−(2)
(C) Items lacking a declaration of gluten or failing to explicitly indicate its presence (21)			
Cereal products	9	+(7)/−(2)	+(7)/−(2)
Oat drinks	3	+(1)/−(2)	+(1)/−(2)
Oat flakes	8	+(2)/−(6)	+(5)/−(3)
Deli products	1	−(1)	−(1)
(D) “gluten free” items (labeled or certified) (14)			
Bakery products	6	−(6)	−(6)
Noodles	1	−(1)	−(1)
Baby preparations	2	−(2)	−(2)
Corn flakes	1	−(1)	−(1)
Oat flakes	1	−(1)	−(1)
Deli products	3	−(3)	−(3)

Minus sign (−) indicates gluten content lower than 20 mg/kg of gluten, which allows labeling with “gluten free” statement according to the European legislation. Plus sign (+) indicates values above the mentioned limit. The gliadin-PWG was used as reference material to calculate the standard curve for the developed ELISA and the R5 sandwich ELISA.

## Data Availability

The original contributions presented in the study are included in the article, further inquiries can be directed to the corresponding author.
